# Design, Synthesis and Cellular Characterization of a Dual Inhibitor of 5-Lipoxygenase and Soluble Epoxide Hydrolase

**DOI:** 10.3390/molecules22010045

**Published:** 2016-12-29

**Authors:** Karin Meirer, Daniel Glatzel, Simon Kretschmer, Sandra K. Wittmann, Markus Hartmann, René Blöcher, Carlo Angioni, Gerd Geisslinger, Dieter Steinhilber, Bettina Hofmann, Robert Fürst, Ewgenij Proschak

**Affiliations:** 1 Institute of Pharmaceutical Chemistry, Goethe-University of Frankfurt, Max-von-Laue Str. 9, D-60438 Frankfurt am Main, Germany; karin.meirer@boehringer-ingelheim.com (K.M.); simkrets@pharmchem.uni-frankfurt.de (S.K.); wittmann@pharmchem.uni-frankfurt.de (S.K.W.); hartmann@pharmchem.uni-frankfurt.de (M.H.); bloecher@ucdavis.edu (R.B.); steinhilber@em.uni-frankfurt.de (D.S.); hofmann@pharmchem.uni-frankfurt.de (B.H.); 2 Institute of Pharmaceutical Biology, Goethe-University of Frankfurt, Max-von-Laue Str. 9, D-60438 Frankfurt am Main, Germany; Glatzel@em.uni-frankfurt.de; 3 Institute of Clinical Pharmacology, Goethe-University of Frankfurt, Theodor-Stern-Kai 7, D-60590 Frankfurt am Main, Germany; angioni@em.uni-frankfurt.de (C.A.); geisslinger@em.uni-frankfurt.de (G.G.)

**Keywords:** soluble epoxide hydrolase, 5-lipoxygenase, inflammation, designed multitarget ligands, leukocyte-endothelial cell interaction

## Abstract

The arachidonic acid cascade is a key player in inflammation, and numerous well-established drugs interfere with this pathway. Previous studies have suggested that simultaneous inhibition of 5-lipoxygenase (5-LO) and soluble epoxide hydrolase (sEH) results in synergistic anti-inflammatory effects. In this study, a novel prototype of a dual 5-LO/sEH inhibitor **KM55** was rationally designed and synthesized. **KM55** was evaluated in enzyme activity assays with recombinant enzymes. Furthermore, activity of **KM55** in human whole blood and endothelial cells was investigated. **KM55** potently inhibited both enzymes in vitro and attenuated the formation of leukotrienes in human whole blood. **KM55** was also tested in a cell function-based assay. The compound significantly inhibited the LPS-induced adhesion of leukocytes to endothelial cells by blocking leukocyte activation.

## 1. Introduction

The arachidonic acid (AA) cascade is one of the main regulatory pathways involved in the inflammatory response and associated diseases. The initial step of the AA cascade is the release of AA from membrane phospholipids by phospholipases [1]. Free AA is subsequently metabolized in three distinct branches of the AA cascade. The cyclooxygenase (COX) branch leads to the formation of various prostaglandins and thromboxane [2]. Leukotrienes and lipoxins are formed by the enzymes of the lipoxygenase (LO) branch [3]. Oxidation of AA by cytochrome P450 (CYP) enzymes results in the biosynthesis of epoxyeicosatrienoic acids (EETs), which are subsequently degraded to dihydroxyeicosatrienoic acids (DHETs) by the enzyme soluble epoxide hydrolase (sEH) [4]. Almost all key enzymes of the AA cascade are of interest in pharmaceutical research, however, only COX inhibitors, one 5-LO inhibitor, and antagonists of cysteinyl leukotriene receptors and thromboxane receptors are in clinical use.

Modulators of the AA cascade not only affect their primary targets; the accumulation of the substrates and regulatory crosstalk between the products leads to pronounced shunting effects. One of the best investigated effects is the accumulation of leukotrienes as a consequence of COX inhibition, leading to analgesic-induced asthma [5]. The shunting phenomena can have beneficial effects; e.g., the inhibition of leukotriene A4 hydrolase leads to increased formation of lipoxin A4, a pro-resolvatory lipid mediator [6,7]. An interesting crosstalk involves the lipoxygenase and CYP branch of the AA cascade. The inhibition of sEH and subsequent accumulation of AA-derived epoxides leads on the one hand to the formation of lipoxins [8] and on the other hand to increased levels of pro-inflammatory LOX products [9]. Most interestingly, the simultaneous inhibition of sEH and 5-lipoxygenase activating protein (FLAP) led to a synergistic anti-inflammatory response in vivo [10].

The described effects support the paradigm change in drug discovery from selective ligands towards designed multi-target ligands (DMLs), which might exhibit improved safety and efficacy [11]. The possibilities and limitations of DMLs in the context of the AA cascade have been excessively reviewed [12,13,14]. Several approaches have been made to design DMLs that inhibit 5-LO and sEH simultaneously. Moser et al. identified a benzimidazole derivative as a dual sEH/5-LO inhibitor using virtual screening [15]. A hybrid imidazo-[1,2*a*]-pyridine-based inhibitor was designed by Meirer et al. and exhibited high potency in vitro [16], but failed to affect cellular systems due to unfavorable plasma stability (data not shown). A fragment-based approach was followed by Achenbach et al. who identified and subsequently optimized a 2-aminothiazole derivative as a dual sEH/5-LO inhibitor [17]. In this study, we present the rational design of a novel hybrid dual sEH/5-LO as a chemical tool for the investigation of cellular effects of the simultaneous inhibition of sEH and 5-LO.

## 2. Results

### 2.1. Design and Synthesis of ***KM55***, a Dual sEH/5-LO Inhibitor

**Scheme 1:**
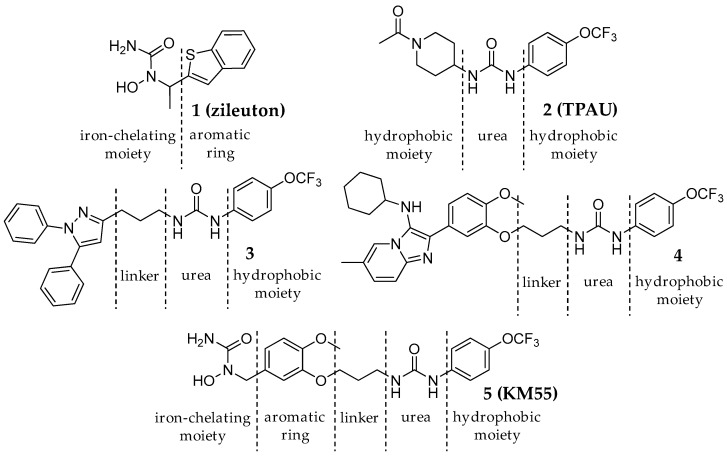
Design strategy for a dual sEH/5-LOX inhibitor.

A critical step in the design of a hybrid DML is the careful choice of pharmacophores for the individual targets and the linker to interconnect them [11]. The only approved inhibitor of 5-LO, zileuton (**1**, Scheme 1), exhibits an iron-chelating moiety, which interacts with the non-heme iron ion in the catalytic center and an aromatic ring [18]. Substituted ureas are well-established epoxide mimetics interacting with the catalytic center of sEH [19]. One of the substituents, a 4-trifluoromethoxy phenyl moiety, was shown to contribute to the high potency and excellent pharmacokinetic profile of a range of sEH inhibitors [20], represented by 1-trifluoromethoxyphenyl-3-(1-acetylpiperidin-4-yl)-urea (TPAU, **2**, Scheme 1). Considering the linker between the two pharmacophores, studies by Meirer et al. [16] and Hwang et al. [21] have shown that a propyl spacer is most suitable. These considerations lead to the design of 1-(3-{5-(hydroxyureido)methyl-2-methoxyphenoxy}propyl)-3-[4-(trifluoromethoxy)phenyl]urea **5** (**KM55**) as a potential dual sEH/5-LO inhibitor.

**Scheme 2:**
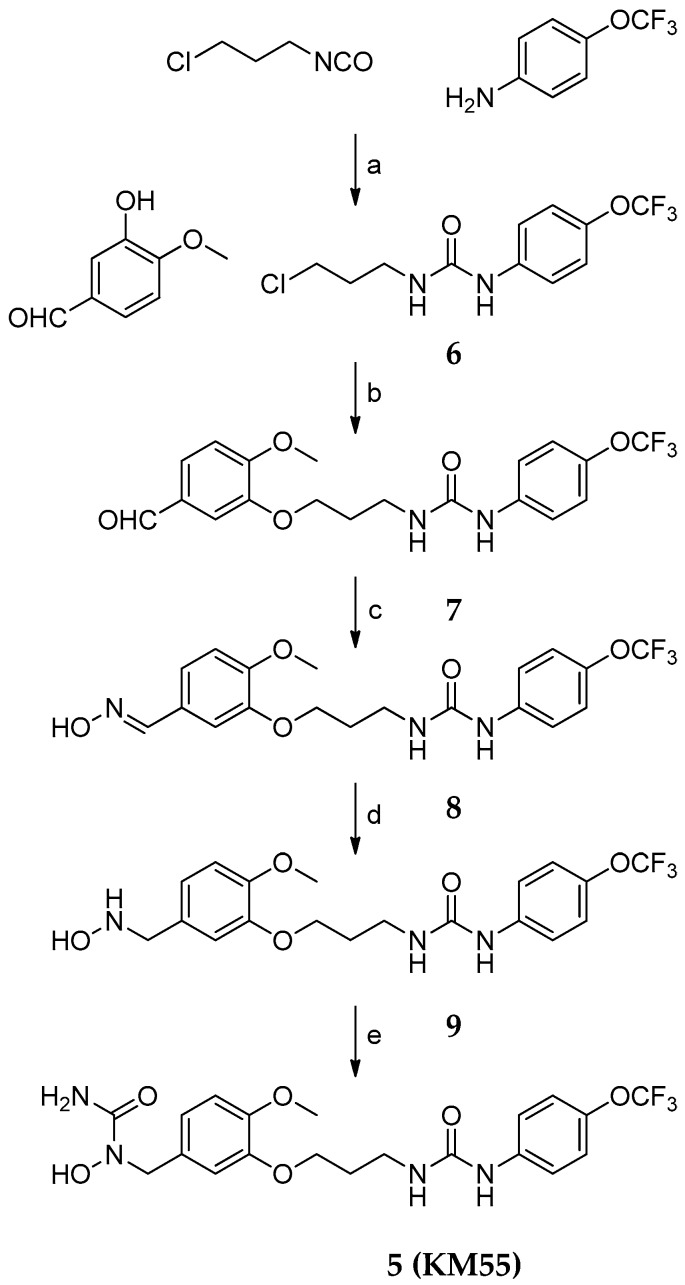
Synthesis of **KM55**. *Reagents and Conditions*: (a) DCM, 0 °C to r.t.; overnight (b) Cs_2_CO_3_, DMF, 70 °C; overnight (c) NH_2_OH *HCl, NaAc, EtOH/MeOH (2:1), H_2_O, r.t., 24 h (d) NaCNBH_3_, methyl orange, HCl, MeOH, THF, argon; r.t. (e) Me_3_SiNCO, dioxane, r.t., 2 h.

The synthesis of **KM55** was accomplished in five linear steps (Scheme 2). First, the urea was formed from 3-chloropropyl isocyanate and 4-(trifluoromethoxy)aniline in dichloromethane. A Williamson ether synthesis procedure was used to couple the linker to 3-hydroxy-4-methoxy-benzaldehyde. The following steps for the introduction of the hydroxyurea moiety were performed according to the procedure described by Malamas et al. [22]. Therefore, the hydroxyimine was formed and subsequently reduced with sodium cyanoborohydride. After the introduction of the terminal hydroxyurea, a purification by reversed-phase HPLC was required to obtain compound **5** (**KM55**) in sufficient purity for biological testing.

### 2.2. In Vitro Evalulation of ***KM55*** on Recombinant Enzymes and in Human Whole Blood

**Figure 1:**
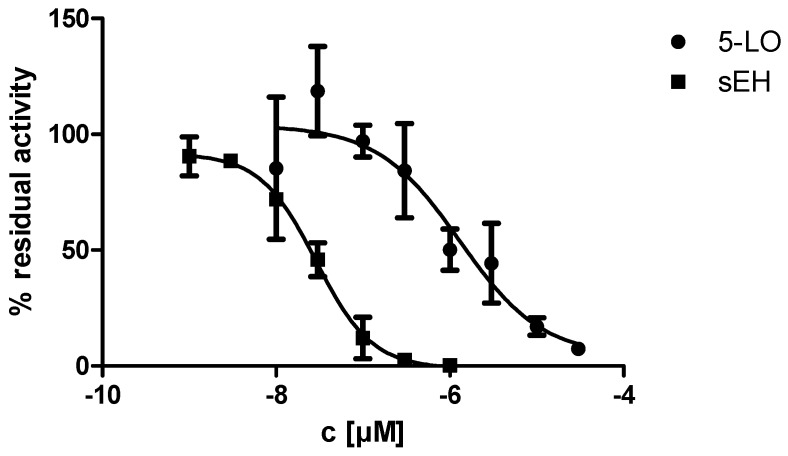
Inhibitory effect of **KM55** on recombinant enzymes.

In order to evaluate the potency of **KM55**, in vitro enzyme activity assays using recombinant proteins were performed. **KM55** potently inhibited sEH in a fluorescence-based activity assay with an IC_50_ of 29 nM (logIC_50_ = −7.5; std. error(logIC_50_) = 0.03) which is comparable to the reference inhibitor *N*-cyclohexyl-*N*′-(4-iodophenyl)urea (CIU) [23] (IC_50_ = 140 nM; logIC_50_ = −6.8; std. error(logIC_50_) = 0.02; Supplementary Materials Figure S1) Recombinant 5-LO activity was measured using the natural substrate arachidonic acid. **KM55** inhibited 5-LO with an IC_50_ of 1.3 µM (logIC_50_ = −5.9; std. error(logIC_50_) = 0.3) (Figure 1) which is in the same range as 5-LO reference inhibitor zileuton (IC_50_ = 650 nM; logIC_50_ = −6.5; std. error(logIC_50_) = 0.13; Figure S2).

**Figure 2:**
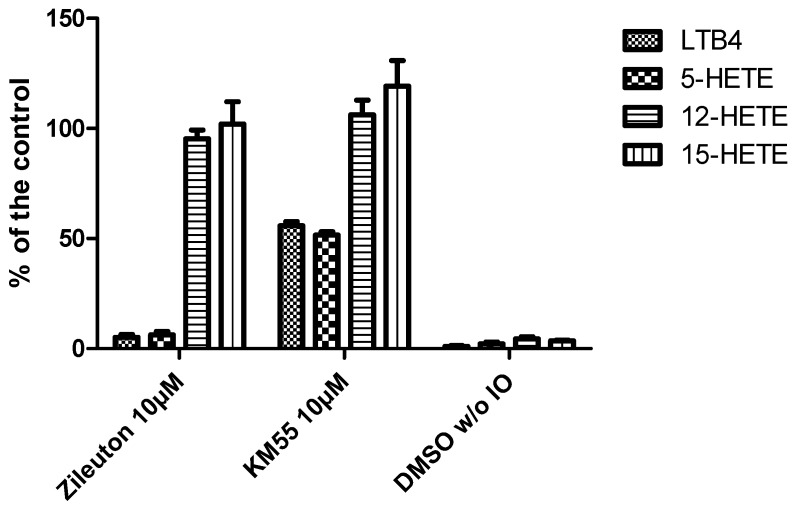
Inhibitory effect of zileuton and **KM55** on lipoxygenase product formation in human whole blood. Data are expressed as mean ± S.E.M. (*n* = 3).

The effect of **KM55** on lipoxygenase product formation was further evaluated in a human whole blood assay. **KM55** inhibited the formation of leukotriene B4 (LTB4) and 5-hydroxyeicosatrienoic acid (5-HETE). The formation of 12-HETE and 15-HETE remained unaffected (Figure 2). 

### 2.3. Effects of ***KM55*** on Leukocyte-Endothelial Cell Interaction

**Figure 3:**
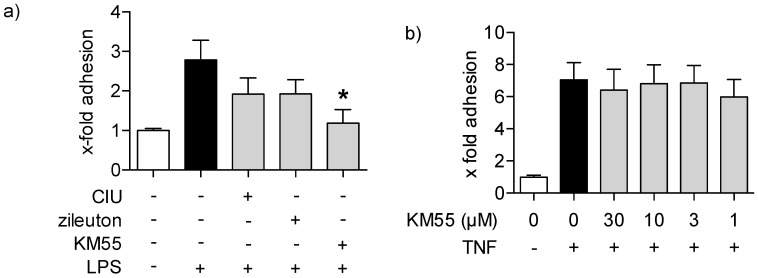
**KM55** blocks leukocyte-endothelial cell interaction by impairing leukocyte activation, whereas endothelial cells are not affected. (**a**) The adhesion of monocyte-like THP-1 cells onto HUVECs is significantly reduced by 30 µM **KM55** (* *p* < 0.05 vs. LPS alone). THP-1 cells where pretreated with CUI (10 µM), zileuton (10 µM) or **KM55** (30 µM, each) for 30 min and were then activated with 1 µg/mL LPS for 24 h; (**b**) HUVECs were pretreated with 30 µM **KM55** for 30 min before they were activated with 10 ng/mL TNF-α for 24 h. THP-1 cells were left untreated. Adhesion assays were performed as described in the section Materials and Methods. Data are expressed as mean ± S.E.M. (*n* = 3).

In order to evaluate the effect of **KM55** in a cell function-based assay, we analyzed the action of the compound on the adhesion of leukocytes to endothelial cells, a key process in acute and chronic inflammation. Two sets of experiments were performed, in which either human leukocytes (monocytic THP-1 cells) or human primary endothelial cells (HUVECs) were treated with **KM55**. Figure 3a shows that **KM55**, which was only applied to leukocytes, strongly inhibited the lipopolysaccharide (LPS)-induced adhesion of the leukocytes to endothelial cells. The 5-LO inhibitor zileuton and the sEH inhibitor CIU were applied for comparison. Used at the same concentration as **KM55**, they only slightly inhibited cell adhesion. In the second setting, in which only endothelial cells were treated with **KM55**, the leukocyte-endothelial cell interaction was not reduced (Figure 3b).

## 3. Discussion

**KM55** is a designed multi-target ligand which inhibits the enzymatic activity of 5-LO and sEH. In the case of **KM55** the combination of the essential pharmacophore features of both enzymes was successful. One of the reasons might be the similarity of the endogenous substrates of 5-LO–arachidonic acid and sEH–EETs, which facilitates the dual activity. The potency of **KM55** is not balanced. The reason for this might be the fact that ureas are transition state mimetics of sEH [24,25], which are commonly known as very potent inhibitors and is comparable to the reference compound AUDA and CIU in our assay, while the inhibitory potency of **KM55** is comparable to that of zileuton [16]. However, in the whole blood setting, **KM55** inhibits about 50% of the 5-LO product formation compared to zileuton, which might result from higher plasma protein binding. 

In this study, we could show that the dual 5-LO/sEH inhibitor **KM55** potently inhibits the adhesion of leukocytes onto endothelial cells by impairing leukocyte function. The adhesive properties of **KM55**-treated monocyte-like THP-1 cells were completely reduced down to control levels. Equimolar concentrations of CIU or zileuton showed a clear, but statistically not significant tendency to also reduce leukocyte adhesion. Thus, the dual inhibitor **KM55** exhibited a stronger action compared to both inhibitors in this cell function-based assay. In contrast, endothelial cells are not affected by **KM55**. This is in line with several studies indicating that the 5-LO pathway plays a minor role or is even not existing in endothelial cells [26,27].

Overall, **KM55** can be regarded as a novel prototype of a dual 5-LO/sEH inhibitor and can be used for cellular investigation and as a starting point for optimization. 

## 4. Materials and Methods

### 4.1. General Information

All reagents and solvents were purchased from the suppliers Sigma-Aldrich (Taufkirchen, Germany), Apollo Scientific (Stockport, UK), Acros Organics (Geel, Belgium) or Alfa Aesar (Karlsruhe, Germany) and were used without further purification. Flash chromatography was performed on packed silica columns (particle size 50 μM) from Varian Medical Systems GmbH (Darmstadt, Germany). NMR spectra were measured on AV 250 nuclear magnetic resonance spectrometer from Bruker (Fällanden, Switzerland). Chemical shifts are reported in parts per million (ppm) using TMS as internal standard; ^1^H (250/300 MHz), ^13^C (63/75 MHz). Mass spectra were measured using ESI with a VG Platform II spectrometer by Fisons (Loughborough, UK) or a Mariner Biospectrometry Workstation by Perspective Biosystems (Waltham, MA, USA). HRMS spectra were measured by a MALDI LTQ Orbitrap XL spectrometer from Thermo Scientific (Waltham, MA, USA). HPLC was performed on a LC2020 system (Shimadzu, Duisburg, Germany), using the following conditions: acetonitrile/water + 0.1% *V*/*V* formic acid gradient run (10%–90% AcN in 10 min, then hold 6 min 90% AcN, 90%–10% in 1 min, hold 10% for 3 min) over 20 min using a Luna 10 μm C18 (2) 100 Å, 250 × 4.6 mm (Phenomenex, Aschaffenburg, Germany) at room temperature, UV detection was set at 254 nm and 280 nm. All compounds were characterized by ^1^H-NMR and ESI MS, final compounds additionally by ^13^C-NMR, HRMS and HPLC to determine purity >95%.

### 4.2. Synthesis

*1-(3-Chloropropyl)-3-[4-(trifluoromethoxy)phenyl]urea* (**6**). 3-Chloropropylisocyanate (1.50 g, 12.55 mmol) was added to a solution of 4-(trifluoromethoxy)aniline (2.22 g, 12.55 mmol) in DCM (50 mL) at 0 °C. The mixture was allowed to warm to room temperature and was stirred overnight. A yellow solution was obtained. The solvent was removed under reduced pressure and the white solid was triturated with hexane to form the pure product **6** (3.14 g, 84% yield). ^1^H-NMR (250 MHz, DMSO-*d*_6_): δ 8.67 (s, 1H), 7.51–7.43 (m, 2H), 7.23–7.18 (m, 2H), 6.32 (t, *J* = 5.77 Hz, 1H), 3.66 (t, *J* = 6.50 Hz, 2H), 3.21 (q, *J* = 7.50 Hz, 2H), 1.88 (quint, *J* = 6.61 Hz, 2H). MS (ESI, 70 eV) *m*/*z* (%): 297.0 (100) (M + H).

*1-[3-(5-Formyl-2-methoxyphenoxy)propyl]-3-[4-(trifluoromethoxy)phenyl]urea* (**7**). 3-Hydroxy-4-methoxy-benzaldehyde (1.54 g, 10.11 mmol) and cesium carbonate (3.29 g, 10.11 mmol) in DMF (40 mL) were heated to 70 °C for 30 min. **6** (3.00 g, 10.11 mmol), dissolved in DMF (40 mL), was added and the mixture was stirred overnight at 70 °C. The solvent was removed under reduced pressure and the residue was diluted with EtOAc (20 mL). The organic phase was extracted with water (20 mL), NaOH solution (1 M, 20 mL twice) and again with water (20 mL). After drying the organic layer over MgSO_4_, the solvent was removed under reduced pressure. The product **7** was recrystallized as a white solid (1.75 g, 42% yield) from hexane/EtOAc. ^1^H-NMR (250 MHz, DMSO-*d*_6_): δ 9.83 (s, 1H), 8.64 (s, 1H), 7.57 (dd, *J*_1_ = 1.81, *J*_2_ *=* 8.25 Hz, 1H), 7.50–7.45 (m, 2H), 7.42–7.39 (m, 1H), 7.30–7.17 (m, 3H), 6.33 (t, *J* = 5.66 Hz, 1H), 4.08 (t, *J* = 6.30 Hz, 2H), 3.88 (s, 3H), 3.29–3.19 (m, 2H), 2.00–1.88 (m, 2H). MS (ESI, 70 eV) *m*/*z* (%): 411.4 (100) (M − H).

*1-(3-{5-(Hydroxyimino)methyl-2-methoxyphenoxy}propyl)-3-[4-(trifluoromethoxy)phenyl]urea* (**8**). **7** (0.50 g, 1.21 mmol) was dissolved in EtOH/MeOH (2:1, 50 mL). Hydroxylamine hydrochloride (0.26 g, 2.43 mmol), sodium acetate (0.40 g, 4.85 mmol) and H_2_O (10 mL) were added to the solution and the mixture was stirred at room temperature for 24 h. Afterwards, the mixture was poured into H_2_O and extracted with EtOAc (100 mL). The organic layer was dried over MgSO_4_. After evaporation of the solvent, the crude product was crystallized from acetone/Et_2_O (0 °C) to give **8** as a white solid (0.50 g, 97% yield). The product was used for the next step without further purification. ^1^H-NMR (250 MHz, DMSO-*d*_6_): δ 10.93 (s, 1H), 8.65 (s, 1H), 8.03 (s, 1H), 7.51–7.44 (m, 2H), 7.24–7.19 (m, 3H), 7.09 (dd, *J*_1_ = 8.30 Hz, *J*_2_ = 1.70 Hz, 1H), 6.98 (d, *J* = 8.30 Hz, 1H), 6.32 (t, *J* = 5.40 Hz, 1H), 4.01 (t, *J* = 6.25 Hz, 2H), 3.78 (s, 3H), 3.31–3.22 (m, 2H), 2.02–1.94 (m, 2H). MS (ESI, 70 eV) *m*/*z* (%): 428.5 (100) (M + H).

*1-(3-{5-(Hydroxyamino)methyl-2-methoxyphenoxy}propyl)-3-[4-(trifluoromethoxy)phenyl]urea* (**9**). Sodium cyanoborohydride (NaCNBH_3_, 0.24 g, 3.74 mmol) in dry THF (10 mL) was added into a solution of **8** (0.50 g, 1.17 mmol) in MeOH (50 mL) under argon atmosphere. Methyl orange was added in order to control the pH of the reaction (*Warning!* HCN develops under acidic conditions!). After 5 min of stirring, a solution of 4 N HCl in dioxane was added dropwise to maintain pH at 3–4. When a steady red color was obtained, the mixture was poured into H_2_O, basified with 1 N NaOH and extracted with EtOAc (2 × 70 mL). The organic layer was dried over MgSO_4_ and the solvent was evaporated under reduced pressure. The product **9** as a white solid (0.50 g, quant. yield) was used for the next step without further purification. ^1^H-NMR (250 MHz, MeOD-*d*_4_): δ 7.46–7.40 (m, 2H), 7.17–7.12 (m, 2H), 7.03 (s, 1H), 6.92–6.90 (m, 2H), 4.11 (t, *J* = 6.04 Hz, 2H), 3.90 (s, 2H), 3.81 (s, 3H), 3.42 (t, *J* = 6.60 Hz, 2H), 2.07–1.96 (m, 2H). MS (ESI, 70 eV) *m*/*z* (%): 428.4 (100) (M − H).

*1-(3-{5-(Hydroxyureido)methyl-2-methoxyphenoxy}propyl)-3-[4-(trifluoromethoxy)phenyl]urea* (**KM55**, **5**). Trimethylsilylisocyanate (0.19 mL, 1.40 mmol) was added into a solution of **9** (0.50 g, 1.16 mmol) in dioxane (30 mL). After stirring for 2 h, the mixture was poured into H_2_O, acidified with 2N HCl and extracted with EtOAc (2 × 30 mL). The organic layer was dried over MgSO_4_ and the solvent was evaporated. Purification of the crude product was accomplished using reversed phase HPLC, yielding **5** as a waxy solid (0.03 g, 5.5%). ^1^H-NMR (250 MHz, CD_3_OD-*d*_4_): δ 7.46–7.40 (m, 2H), 7.16–7.12 (m, 2H), 6.99 (bs, 1H) 6.93–6.87 (m, 2H), 4.54 (s, 2H), 4.09 (t, *J* = 6.09 Hz, 2H), 3.81 (s, 3H), 3.41 (t, *J* = 6.62 Hz, 2H), 2.01 (quint, *J* = 6.35 Hz, 2H). MS (ESI, 70 eV) *m*/*z* (%): 473.9 (100) (M + H). ^13^C-NMR (75 MHz, CD_3_OD-*d*_4_): δ 164.1, 158.1, 150.4, 149.6, 145.1, 140.3, 131.6, 123.7, 122.7, 122.6, 121.0, 115.5, 113.1, 68.2, 56.6, 54.4, 38.2, 30.8. HRMS calculated *m*/*z* 495.14674, found *m*/*z* 495.14619. HPLC: *t_R_*: 9.4 min, purity >95%.

### 4.3. Recombinant sEH Enzyme Activity Assay

The IC_50_ values of the compounds were determined by a fluorescence-based assay system of 96-well format. As substrate non-fluorescent 3-phenyl-cyano-(6-methoxy-2-naphthalenyl)methyl ester-2-oxirane-acetic acid (PHOME, Cayman Chemicals, Ann Arbor, MI, USA) was used, which can be hydrolyzed by the sEH to the fluorescent 6-methoxynaphtaldehyde [28]. The formation of the product was measured (λ_em_ = 330 nm, λ_ex_ = 465 nm) by a Tecan Infinite^®^ F200 Pro plate reader (Tecan Trading AG, Männedorf, Switzerland). Therefore, recombinant human sEH (2 μg per well) in Bis-Tris buffer pH 7 with 0.1 mg·mL^−1^ BSA containing a final concentration of 0.01% Triton-X 100. 100 μL of protein solution were incubated with different concentrations of compounds (DMSO with final concentration of 1%) for 30 min. at room temperature. After that 10 μL of substrate were added (final concentration 50 μM). The hydrolysed substrate was measured for 30 min (one point every minute). A blank control (no protein and no compound) as well as a positive control (no compound) was executed.

### 4.4. Recombinant 5-LO Activity Assay

5-LO was purified as described by Kretschmer et al. [29]. In brief, r5-LO was expressed in a 2 × 500 mL culture (*E. coli* BL21(DE3)) and purified in an ÄKTA Xpress system (GE Healthcare, Uppsala, Sweden) using ATP affinity (5 mL ATP-agarose column) and anion exchange chromatography (ResourceQ 1 mL IEX column, GE Healthcare). Three µg of r5-LO were then pre-incubated 15 min at 4 °C with test compound or vehicle control in 1 mL PBS pH 7.4 containing 1 mM EDTA. After 30 s pre-warming at 37 °C, the reaction was started by addition of 2 mM CaCl_2_ and 20 µM AA. The reaction was stopped after 10 min by addition of 1 mL methanol. Prostaglandin B1 (200 ng, internal standard), HCl (1 N, 30 μL), and PBS (500 μL) were added for solid phase extraction. HPLC analysis was performed according to PMC42826 and 2555374. Analysis included leukotriene (LT) B4, its all-*trans* isomers, and 5-hydro(pero)xy-6,8,11,14(*E*,*Z*,*Z*,*Z*)-eicosatetraenoic acid (5-H(P)ETE). Data of three independent measurements was normalized on vehicle control and means ± SEM or IC_50_ values were calculated. For this a nonlinear fit (log (inhibitor) vs. normalized response—variable slope) by GraphPad Prism software version 7.00 for Windows (GraphPad Software, La Jolla, CA, USA) was used.

### 4.5. Human Whole Blood Assay

HWB and leukocyte concentrates were taken with informed consent. In vitro human whole blood assays were performed as described by Maier et al. [30]. In brief, heparinized venous blood was first pre-warmed for 30 min, then pre-incubated with the test compound or vehicle control for 30 min at 37 °C. Reaction was started by addition of 20 µM calcium ionophore (IO) A23187 (final concentration, previously dissolved in 50 μL autologous plasma). After 15 min the reaction was stopped on ice and plasma supernatants were taken after centrifugation at 9391× *g*. Liquid-liquid extraction and LC-MS/MS analysis for LTB_4_ and 5-HETE was performed according to [7]. Data of three independent measurements was normalized to vehicle control (DMSO) and means ± SEM were calculated.

### 4.6. Cell Culture

THP-1 cells (ACC-16) were obtained from the Leibniz Institute DMSZ—German Collection of Microorganisms and Cell Cultures (Braunschweig, Germany) and were cultured in HyClone™ RPMI 1640 media (GE Healthcare, Chicago, IL, USA) containing 10% fetal bovine serum (FBS, Biochrom, Berlin, Germany), 100 U/mL penicillin (PAA, Pasching, Austria) and 100 µg/mL streptomycin (PAA) at 37 °C in an atmosphere with 5% CO_2_. Primary human umbilical vein endothelial cells (HUVECs) were obtained from Provitro (Berlin, Germany) and cultured in Endothelial Cell Growth Medium (PELOBiotech, Planegg, Germany) containing 10% FBS, 100 U/mL penicillin, 100 µg/mL streptomycin and 2.5 µg/mL amphotericin B (PAN-Biotech, Aidenbach, Germany). HUVECs were cultivated until passage 2 and used for experiments at passage 3. Both cell types were cultured at 37 °C in an atmosphere with 5% CO_2_.

### 4.7. Cell Adhesion Assay

HUVECs were seeded in 24-well plates and were grown to confluence. HUVECs and THP-1 were treated as indicated in the respective figure legend. After 30 min pretreatment with the respective compound, either HUVECs were treated with 10 ng/mL TNF-α (PeproTech, Hamburg, Germany) or THP-1 cells were treated with 1 µg/mL LPS (Sigma-Aldrich, Taufkirchen, Germany) for 24 h. THP-1 cells were labelled with Cell-Tracker™ (10 µM; Life Technologies, Carlsbad, CA, USA). 200,000 THP-1 cells were seeded onto HUVEC monolayers and were allowed to adhere for 20 min. After a washing step, which removes non-adherent THP-1 cells, cells were lysed. Fluorescence of the lysates, which correlates with the number of adhered THP-1 cells, was finally measured in a Tecan Infinite^®^ F200 plate reader (Tecan, Männedorf, Switzerland) at 535 nm.

## Supplementary Materials

Supplementary materials can be accessed at: http://www.mdpi.com/1420-3049/22/1/45/s1.
