# Supplementary material for: Design, Synthesis and Cellular Characterization of a Dual Inhibitor of 5-Lipoxygenase and Soluble Epoxide Hydrolase
Source: Molecules. 2016 Dec 29;22(1):45. doi: 10.3390/molecules22010045 (PMC6155600; doi:10.3390/molecules22010045)
Supplement: Supplementary file 1 [file molecules-22-00045-s001.pdf]

Supplementary Materials: Design, Synthesis and Cellular Characterization of a Dual Inhibitor of 5-Lipoxygenase and Soluble Epoxide Hydrolase

Karin Meirer, Daniel Glatzel, Simon Kretschmer, Sandra K. Wittmann, Markus Hartmann, René Blöcher, Carlo Angioni, Gerd Geisslinger, Dieter Steinhilber, Bettina Hofmann, Robert Fürst and Ewgenij Proschak

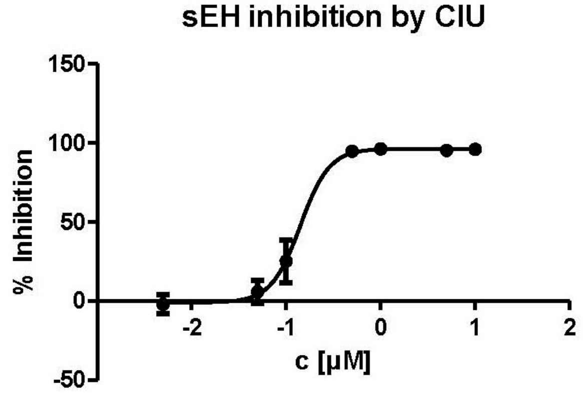

Figure S1. Inhibition of soluble epoxide hydrolase by reference inhibitor *N*-cyclohexyl-*N*′-(4-iodophenyl)urea (CIU).

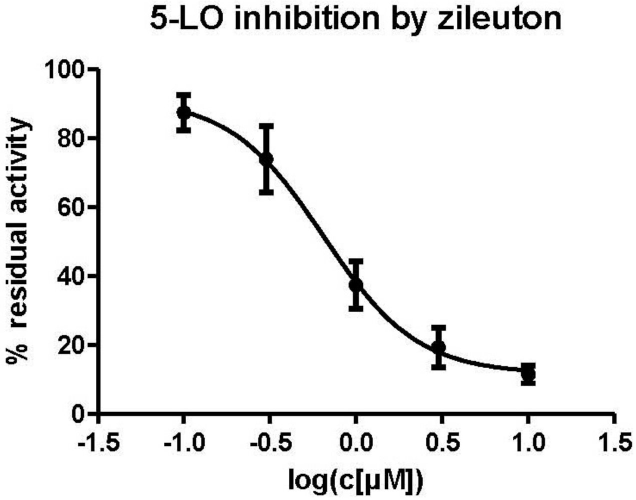

Figure S2. Inhibition of 5-lipoxygenase by reference inhibitor zileuton.

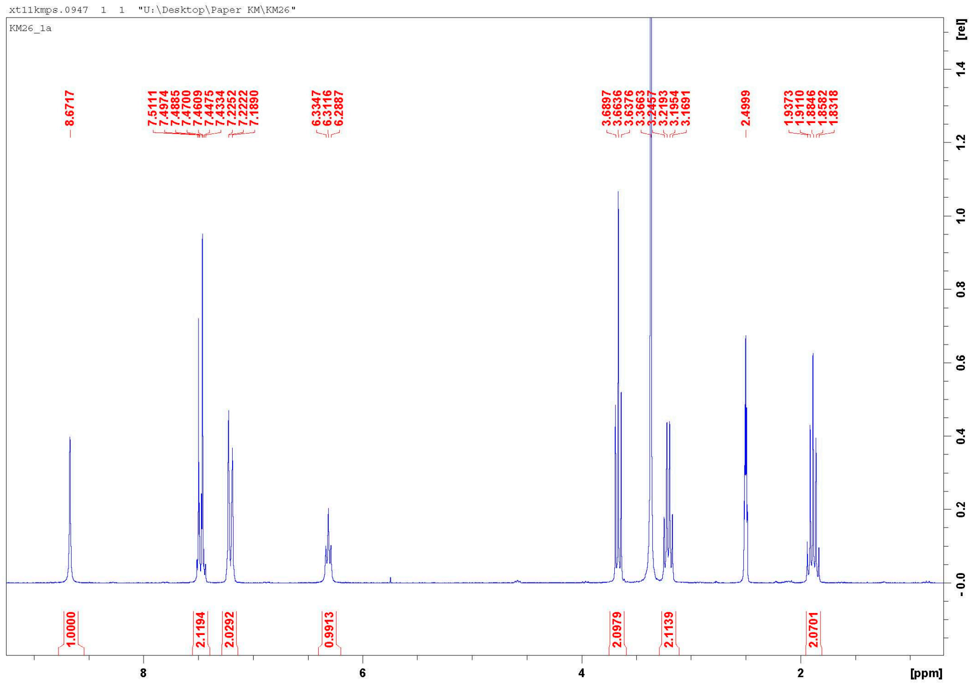

Figure S3. ^{1}H-NMR spectrum of compound **6**.

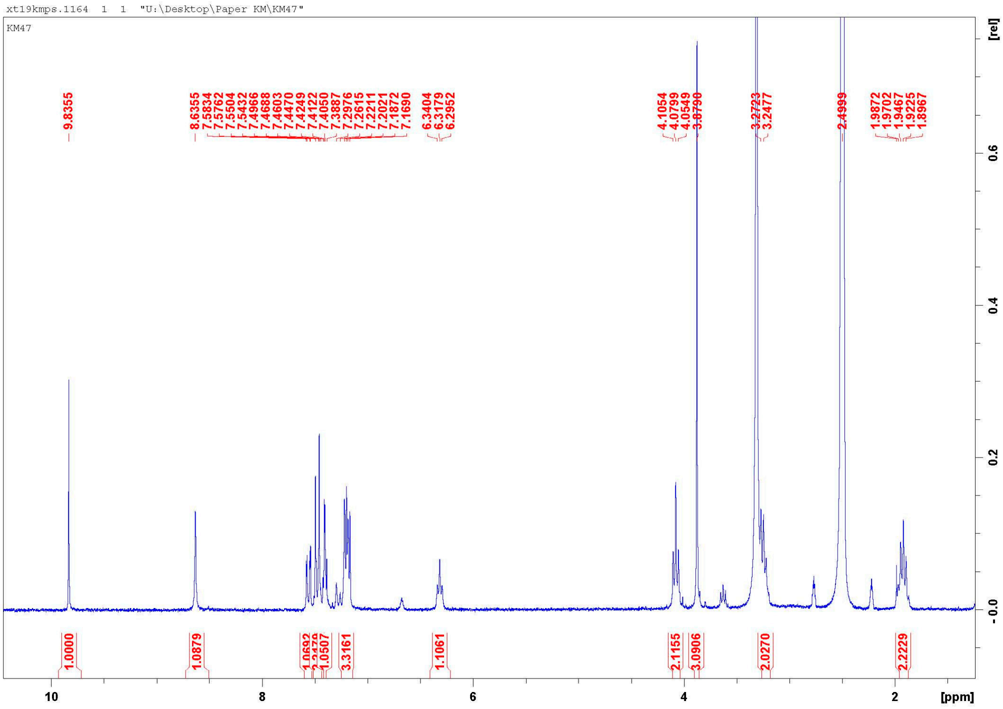

Figure S4. ^{1}H-NMR spectrum of compound **7**.

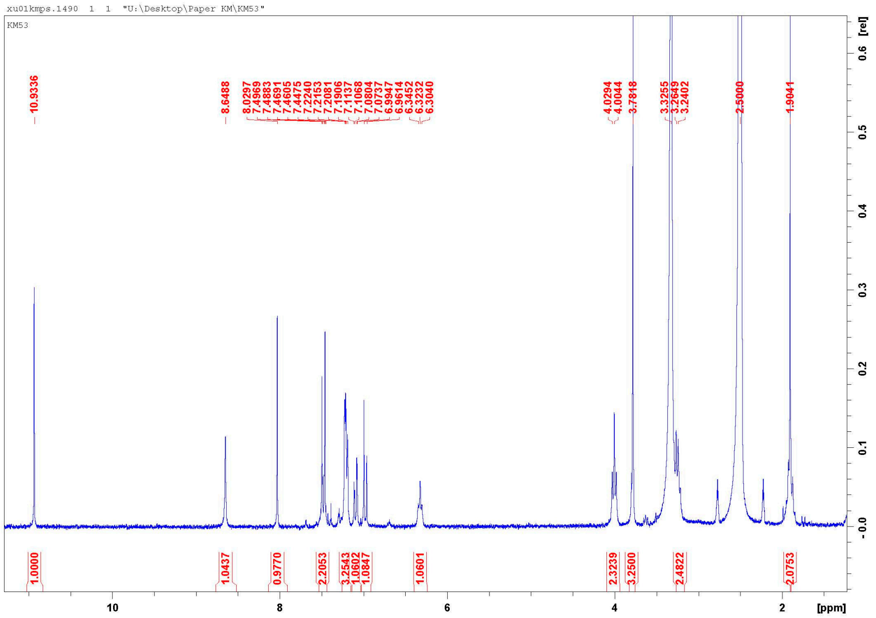

Figure S5. ^{1}H-NMR spectrum of compound **8**.

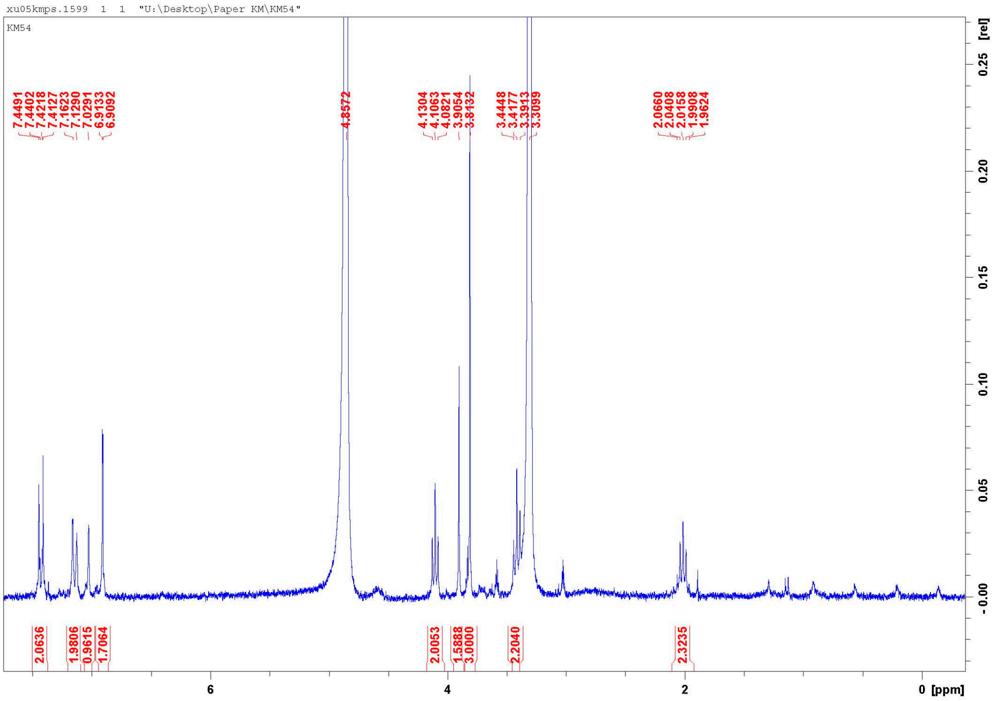

Figure S6. ^{1}H-NMR spectrum of compound **9**.

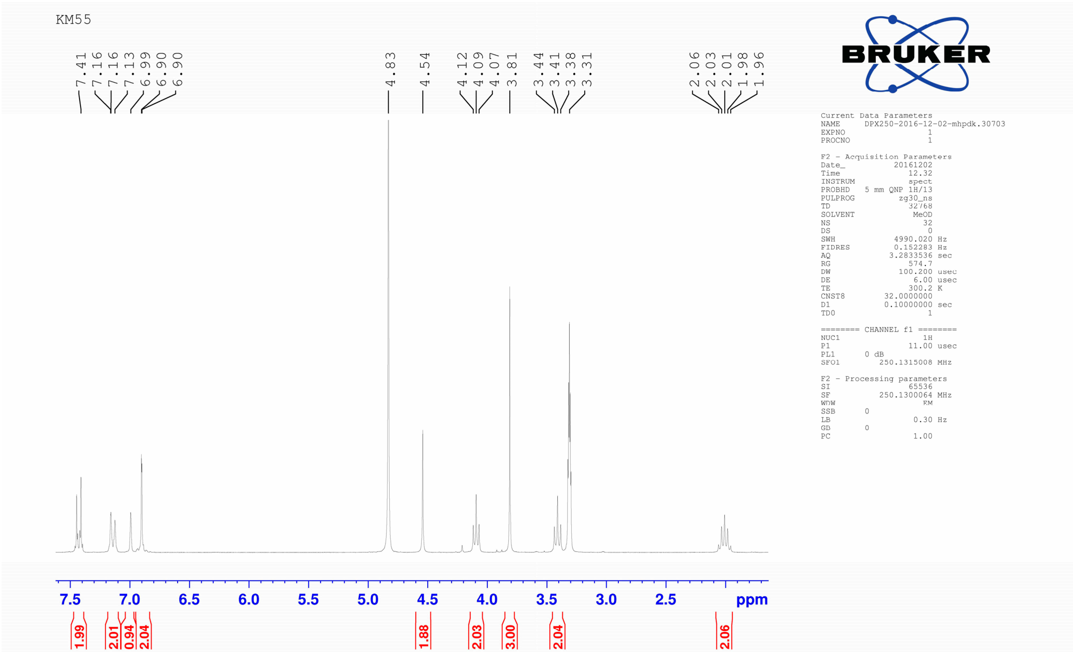

Figure S7. ^{1}H-NMR spectrum of **KM55**.

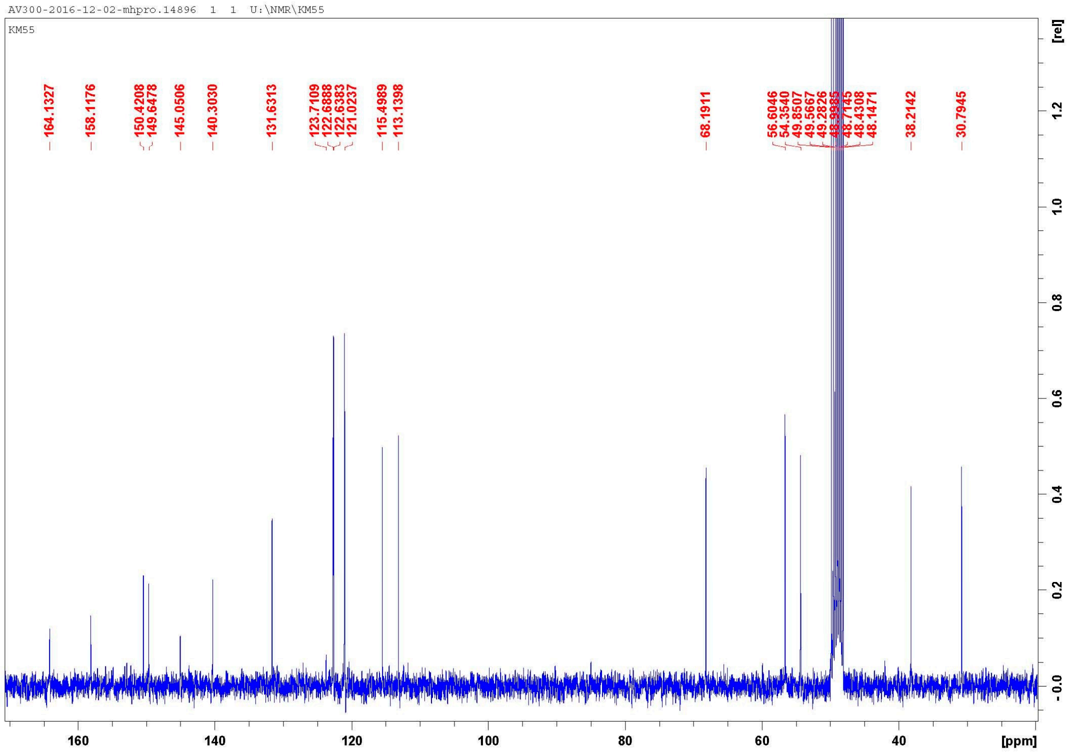

Figure S8. ^{13}C-NMR spectrum of **KM55**.

SHIMADZU LabSolutions **Analysis Report**

<Sample Information>

Sample Name	: KM55		
Sample ID	:		
Data Filename	: KM55_C18_Scout_Theresa_Reinheitsbestimmung_7.lcd		
Method Filename	: C18_Scout_Theresa_Reinheitsbestimmung.lcm		
Batch Filename	: KM55_Reinheit.lcb		
Vial #	: 1-6	Sample Type	: Unknown
Injection Volume	: 20 uL		
Date Acquired	: 29.11.2016 16:34:38	Acquired by	: System Administrator
Date Processed	: 29.11.2016 16:54:39	Processed by	: System Administrator

<Chromatogram>

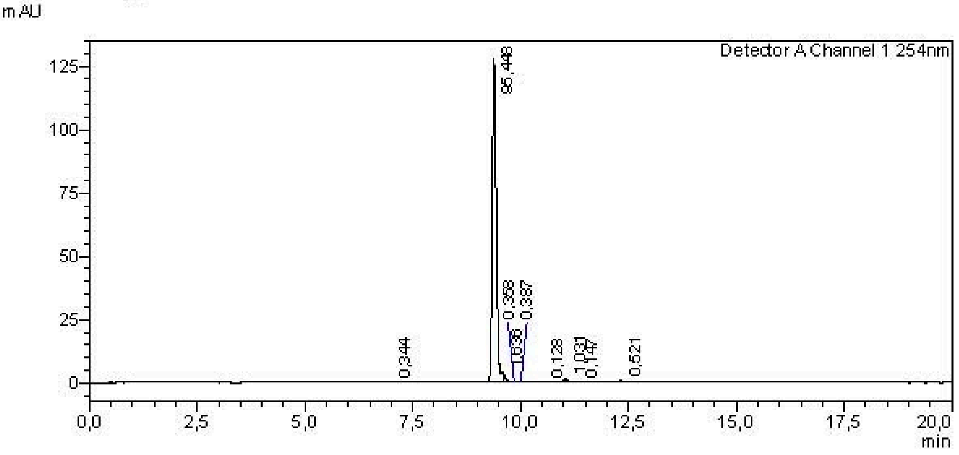

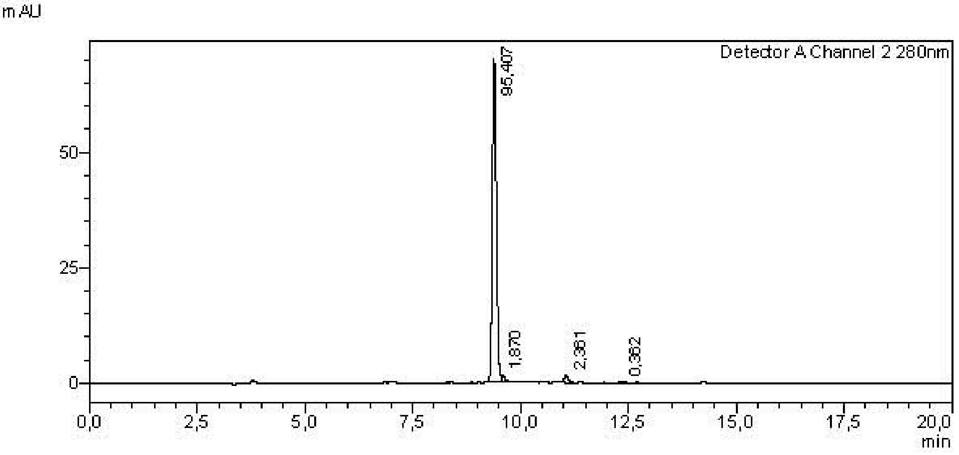

Peak Table

Detector A Channel 1 254nm

Peak#	Ret. Time	Area%
1	7,020	0,344
2	9,386	95,448

Peak#	Ret. Time	Area%
3	9,596	1,636
4	9,851	0,358
5	10,010	0,387
6	10,560	0,128
7	11,055	1,031
8	11,342	0,147
9	12,340	0,521
Total		100,000

Detector A Channel 2 280nm

Peak#	Ret. Time	Area%
1	9,387	95,407
2	9,563	1,870
3	11,055	2,361
4	12,343	0,362
Total		100,000

Figure S9. Purity determination of **KM55**.
